# Bioinformatics and experimental analysis revealed the cancer-promoting role of NCAPG2 in epithelial ovarian cancer

**DOI:** 10.3389/fonc.2026.1574236

**Published:** 2026-03-13

**Authors:** Xiabing Li, Yaping Wang, Luyao Kang, Hai Zhu, Qing Liu, Hongyu Li

**Affiliations:** 1 Gynecologic Oncology, the Third Affiliated Hospital of Zhengzhou University, Zhengzhou, Henan, China; 2 Gynecologic Oncology, Zhengzhou Key Laboratory of Gynecological Oncology, Zhengzhou, Henan, China; 3 Gynecologic Oncology, The International Joint Laboratory of Ovarian Malignant Tumor, Zhengzhou, Henan, China

**Keywords:** bioinformatics analysis, cell metastasis, cell proliferation, epithelial ovarian cancer, NCAPG2

## Abstract

**Introduction:**

Ovarian cancer (OC) is one of the most common gynecological malignancies with an extremely poor prognosis. Among them, epithelial ovarian cancer (EOC) is the most common histological type and exhibits more aggressive behavior. Non-SMC condensin II complex subunit G2 (NCAPG2) is crucial for the execution of chromosomal mitosis and the promotion of tumorigenesis, but its role in the progression of EOC remains unclear.

**Method:**

In this study, we first analyzed the expression of NCAPG2 in EOC using data from the cancer genome atlas program (TCGA) database, Genotype-Tissue Expression (GTEx) project, and the Gene Expression Omnibus (GEO) dataset (GSE9891). Subsequently, bioinformatics tools were used to explore the expression of NCAPG2 in EOC and its related functions. In addition, we also evaluated the role of NCAPG2 in DNA damage repair, chemotherapy resistance, immune cell infiltration, and immunotherapy response. Finally, its expression and function have been verified through clinical samples and *in vitro* experiments.

**Result:**

Analyses of databases revealed that NCAPG2 is significantly overexpressed in EOC tissues and cells. NCAPG2 plays a role in DNA damage repair, chemotherapy resistance, immune cell infiltration, and immunotherapy response. More importantly, knockdown of NCAPG2 by siRNA can inhibit the proliferation, migration, and invasion abilities of EOC cells A2780 and OVCAR3 *in vitro* through the epithelial-mesenchymal transition (EMT) signaling pathway. Clinical specimens have confirmed that NCAPG2 is highly expressed in EOC tissues and is closely related to the clinical stage.

**Conclusion:**

High expression of NCAPG2 can promote the progression of EOC and may serve as a potential novel therapeutic target for EOC.

## Introduction

In women, ovarian cancer (OC) is one of the deadliest cancers (1). Due to the characteristics of the disease, OC lacks early clinical symptoms and has a relatively subtle onset. It is frequently diagnosed at an advanced stage (2, 3). As a result, the survival rate of OC patients is only 30%, indicating a poor prognosis (4). OC primarily includes sex cord-stromal ovarian cancer, germ cell ovarian cancer, and epithelial ovarian cancer (EOC). The most common histological type is EOC (5, 6), which accounts for over 95% of ovarian malignancies (6, 7). EOC has a much worse prognosis and is more aggressive than non-epithelial ovarian cancer (8). The high rate of recurrence and the development of chemoresistance remain the major obstacles to improving patient survival (9), and these clinical challenges are often driven by underlying biological processes such as chromosomal instability and dysregulated cell cycle progression, which are hallmark features of aggressive EOC (10, 11). Therefore, clarifying the key molecular drivers of these processes is of great significance for discovering new therapeutic targets of EOC.

Non-SMC condensin II complex subunit G2 (NCAPG2) is component of the condensing II complex and is crucial for chromosome condensation and segregation during mitosis (12). Studies have shown that NCAPG2 is an important player in the centromere localization of Polo-like kinase 1 (PLK1), because NCAPG2 interacts with PLK1 during the prophase and metaphase of mitosis (13). NCAPG2 localization and PLK1 recruitment to kinetochores of misaligned chromosomes are essential for proper chromosome alignment. PLK1 is a well-established mitotic regulator with multiple biological functions throughout the cell cycle (14). These findings suggest that NCAPG2 may play a critical role in maintaining cancer cell homeostasis and may be a promising target for cancer therapy due to its ability to regulate mitotic chromatin condensation.

NCAPG2 has been shown to facilitate tumorigenesis by contributing to the cell cycle, proliferation, and metastasis in multiple cancers, such as hepatocellular carcinoma (HCC), glioblastoma, lung adenocarcinoma, and malignant melanoma (15–18). In HCC, NCAPG2 overexpression was found, and its high expression was associated with poor clinical outcomes. Enforced expression of NCAPG2 promotes cell proliferation, migration, and invasion in HCC through activation of the signal transducer and activator of transcription 3 (STAT3) and NF-κB signaling pathways (15). In glioblastoma, studies have shown that NCAPG2 expression is significantly increased in tumor tissues and correlates with poor clinical outcomes. In addition, NCAPG2 promotes proliferation, migration, and invasion of glioblastoma cells and regulates the cell cycle. Molecular mechanistic studies suggest that NCAPG2 regulates HBO1 phosphorylation and H4 histone acetyltransferase activation. It also activates of Wnt/β-catenin pathway and facilitates the binding of minichromosome maintenance proteins proteins to chromatin to exert its effects (16). In lung adenocarcinoma, increased NCAPG2 expression regulates cell proliferation and has been identified as a poor prognostic biomarker (17). In malignant melanoma, NCAPG2 drives tumor progression through activation of STAT3 and may be a potential therapeutic target for its treatment (18). However, it remains unclear whether NCAPG2 plays a similar role in EOC.

**Figure 1:**
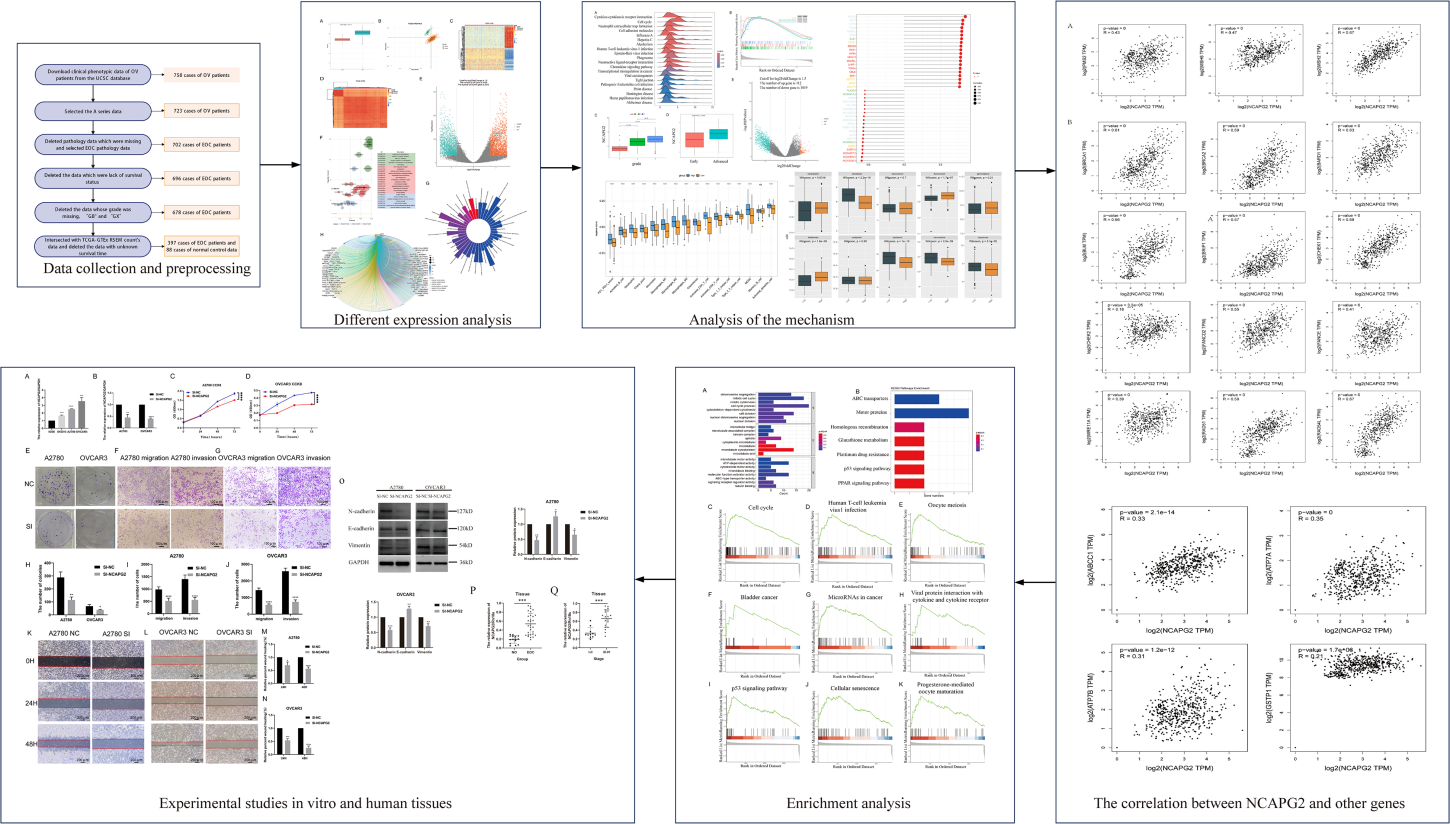
Study design flowchart.

To better understand the predictive value and molecular mechanisms of NCAPG2 in tumor biology, which could improve clinical diagnosis and treatment strategies, public database analysis offers the most efficient approach (19–22). Subsequently, we analyzed NCAPG2 expression in EOC across different stages and grades using data from the cancer genome atlas program (TCGA), the Genotype-Tissue Expression (GTEx) database, and GSE9891. The study design is illustrated in **Figure 1**. Furthermore, we performed functional enrichment and immune infiltration analysis of NCAPG2 and explored the drug-gene associations in EOC. In addition, a series of *in vitro* experiments was conducted to investigate the effects of NCAPG2 downregulation on the occurrence and progression of EOC. Altogether, our findings provide evidence that NCAPG2 is a promising biomarker for EOC.

## 2 Materials and methods

### 2.1 Data collection and preprocessing

**Figure 2:**
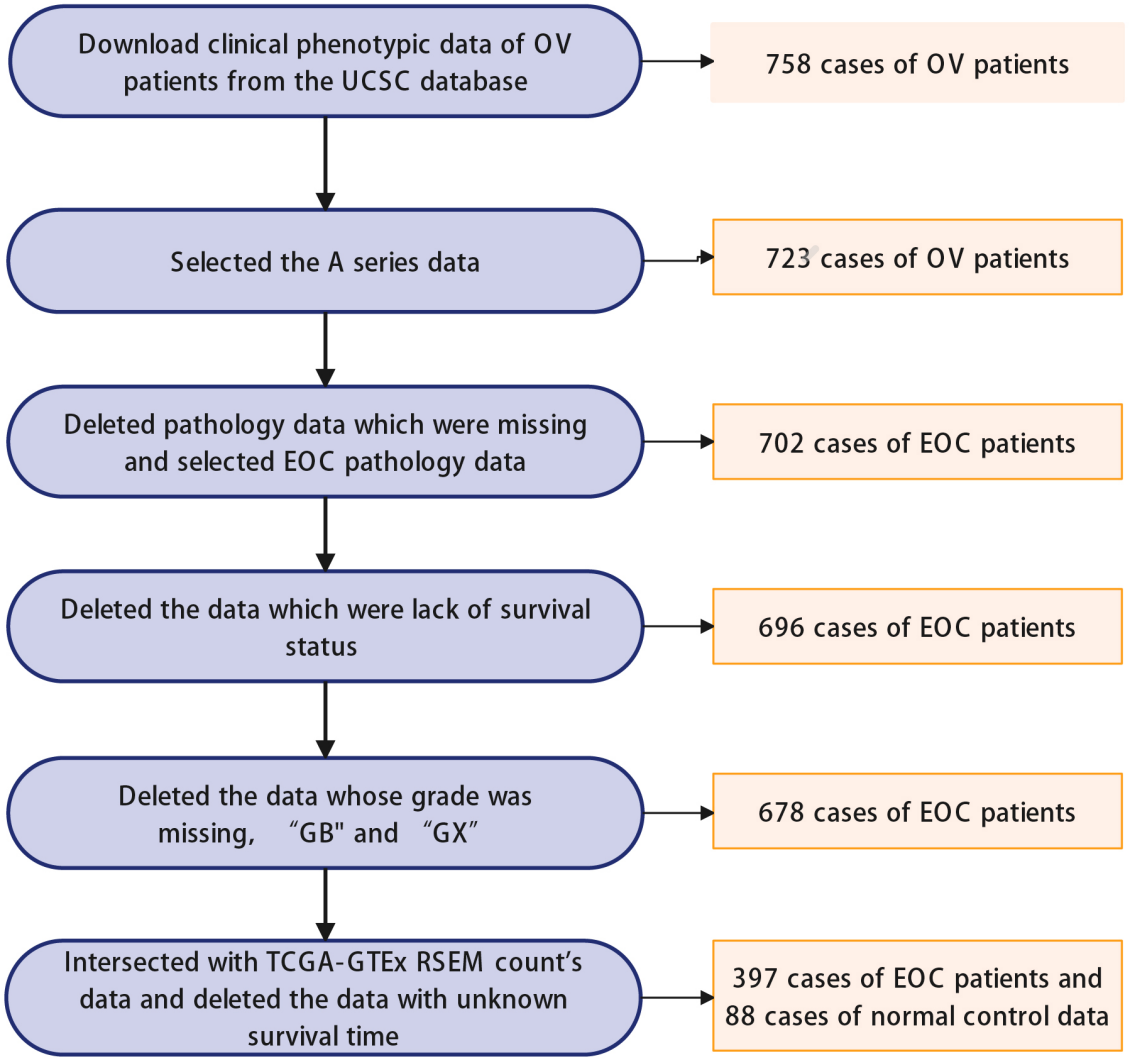
Data screening flowchart: OV: ovarian cancer; EOC: epithelial ovarian cancer.

We downloaded RNA expression and clinical data of OC of the TCGA (23) (https://portal.gdc.cancer.gov) and GTEx (24) (https://www.gtexportal.org/home) through the UCSC-Xena database (http://xenabroswer.net/hub). The data of the TCGA target GTEx for the ovary were used for the following analysis. The final data consisted of 397 EOC patients and 88 normal control cases (normal ovaries), and the detailed information was summarized in **Supplementary Table 1** (**Supplementary Table 1**). The data screening process is shown in **Figure 2**. Dataset named GSE9891 (n=285) was sourced from the Gene Expression Omnibus (GEO) database (https://www.ncbi.nlm.nih.gov/geo/) and was also used for the exploration of the EOC (25).

### 2.2 Different expression analysis

Differential expression analysis of NCAPG2 mRNA levels between EOC tissues and normal ovarian tissues was performed using R (version 4.3.2). Additionally, the association of NCAPG2 expression with different tumor stages, including clinical stage and histological grade, was analyzed. Based on the median NCAPG2 expression level, the data were divided into high and low NCAPG2 groups. Differentially expressed genes (DEGs) between EOC tissues and normal tissues, as well as between the high and low expression groups, were identified using DESeq2 R package (version 1.42.0) (26). Genes with adjusted p-value<10^e-32^ and absolute log2FoldChange greater than 1.5 were considered statistically significant and were further analyzed. The volcano plot was used to visualize the DEGs. DESeq2 was also used to perform the variance stabilizing transformation (vst) of count data prior to differential analysis and downstream analyses.

### 2.3 Correlation expression analysis

Weighted correlation network analysis (WGCNA) is commonly used to identify modules of highly correlated genes. It can summarize these modules by using module trait genes or intra-module hub genes. Additionally, WGCNA employs gene network method to associate modules with each other and with external sample characteristics, and to calculate module membership metrics (27). LinkET can be used to visualize the results of the Mantel test. Using WGCNA (version 1.72-1) and linkET (version 0.0.7.4), we calculated the Pearson correlation coefficient between NCAPG2 and other genes. We selected genes with an absolute Pearson correlation coefficient > 0.5 and p-value < 0.05 as the criteria for further analysis.

### 2.4 Enrichment analysis

Gene Ontology (GO), a bioinformatics tool, was used for gene annotation and analysis of biological processes. The STRING database can not only analyze the interactions between proteins but also provide specific information about individual proteins and offer GO-enrichment functional analysis of related proteins. The Kyoto Encyclopedia of Genes and Genomes (KEGG) comprises biological pathways, genomes, and enzymatic pathways (28). Genes were selected based on differential expression analysis between the NCAPG2-high expression group and the NCAPG2-low expression group, in combination with correlation expression analysis results. The GO (29) terms are grouped into three categories: biological processes (BP), cellular component (CC), and molecular function (MF). Using all the DEGs, Gene Set Enrichment Analysis (GSEA) was also performed to explore functions at the gene set level. ClusterProfiler (version 4.10.0) was used for the analyses above.

### 2.5 Immune cell infiltration analysis

The 184 immune cell signatures were obtained from the work of Shiyuan Wang et al (30). Single-sample gene set enrichment analysis (ssGSEA) using GSVA (version 1.50.0) was performed on TCGA-GTEx data, followed by immune infiltration analysis. A survival analysis package was used to determine the cutoff point based on NCAPG2 expression, and samples were divided into high and low expression groups accordingly.

### 2.6 Drug sensitivity analysis

To analyze the drug sensitivity of drugs potentially targeting NCAPG2, we examined the correlation between NCAPG2 expression levels and drug sensitivity, measured by the 50% inhibitory concentration (IC50), for 10 small molecule drugs using TCGA-GTEx data and oncoPredict (version 0.2) (31). The sample grouping used in this analysis is the same as that applied in the immune cell infiltration analysis.

### 2.7 Bioinformatic analysis of correlation with DNA repair genes and chemotherapy resistance-related genes

The correlation between NCAPG2 mRNA expression and the expression of DNA damage repair (DDR) genes and chemotherapy resistance-related genes was analyzed using the Gene Expression Profiling Interactive Analysis (GEPIA2) web server (http://gepia2.cancer-pku.cn/). This platform is based on data from TCGA and the GTEx projects. The analysis was performed on the OV dataset. A list of key genes involved in mismatch repair (MMR) including PMS2, MSH6, and MSH2); homologous recombination repair (HRR) genes including BRCA1, BRCA2, BARD1, BLM, BRIP1, CHEK1, CHEK2, FANCD2, FANCE, MRE11A, RAD51, and RAD54L); and chemotherapy resistance-related genes including ABCC1 (MRP1), ATP7A, ATP7B, and GSTP1 was compiled. Pearson correlation analysis was performed between NCAPG2 mRNA expression and each gene. The Pearson correlation coefficient (*R* value) and corresponding statistical significance (*P* value) were calculated and displayed by the GEPIA2 platform.

### 2.8 Cell culture

Human EOC cell lines SKOV3, A2780, OVCAR3, and the normal human ovarian epithelial cell line IOSE-80 were provided by the Cell Resource Center, Institute of Basic Medical Sciences, CAMS/PUMC. All cells were cultured in RPMI-1640 medium (Servicebio, Wuhan, China) supplemented with 10% fetal bovine serum (FBS; Sigma, USA) and 1% penicillin-streptomycin (Servicebio, Wuhan, China). The cells were maintained in a 5% CO_2_ incubator at 37 °C. Depending on the rate of cell growth, the culture medium was changed every 1–2 days. Specifically, 4 ml of medium was used in T25 culture flasks, and 7 ml in 10 cm dishes. When the cell density reached approximately 90%, cell passaging was performed. The culture medium was discarded, and the cells were digested with 2 ml of trypsin for 2–3 minutes. Digestion was stopped by adding an equal volume of culture medium. The cell suspension was transferred to a centrifuge tube and centrifuged at 1000 rpm for 5 minutes. After discarding the supernatant, the cell pellet was resuspended in culture medium. The cells were then divided into two portions, seeded into new culture flasks or dishes, supplemented with sufficient medium, mixed gently, and returned to the incubator. The cells attached to the surface within approximately 6–8 hours, after which subsequent experiments could be conducted.

### 2.9 qRT-PCR

qRT-PCR was performed on a total of 15 normal ovarian tissues, 30 EOC tissues, IOSE-80, SKOV3, A2780, and OVCAR3 cells. We isolated and extracted total RNA using RNAiso Plus (Cwbio, Beijing, China) and reverse transcribed the isolated RNA into cDNA using the ReverTra Ace qPCR RT Kit (TOYOBO, Japan). We used the SYBR Green PCR Kit (Cwbio, Beijing, China) for real-time PCR. GAPDH was used as the internal control. The qRT-PCR assay was conducted on a real-time PCR instrument. We used the 2^^-ΔΔCt^ method to analyze and compare the results. The reaction conditions of qRT-PCR are shown in **Supplementary Tables 2**, **3**. And the primer sequences of NCAPG2 and GAPDH are shown in **Supplementary Table 4**.

### 2.10 Transfection

According to the manufacturer’s protocol, SI-NC and SI-NCAPG2 were transfected into OVCAR3 and A2780 cells using Lipofectamine 3000 transfection reagent (Thermo Fisher, USA). The sequences of the siRNAs of SI-NCAPG2 and SI-NC are shown in **Supplementary Table 4**. The specific experimental steps were as follows: When the cell confluency reached 80%, 250 μL serum-free medium and 2.5 μL siRNA were added into a microcentrifuge tube to prepare a siRNA solution. Separately, 250 μL serum-free medium and 1 μL transfection reagent were added into another microcentrifuge tube and incubated at room temperature for 5 minutes. The siRNA solution was then added to the transfection reagent mixture, and the combined solution was mixed gently and incubated at room temperature for 20 minutes to form siRNA-transfection reagent complexes. Each well of cells in a six-well plate was replaced with 500 μL serum-free medium, followed by the addition of 500 μL of the siRNA-transfection reagent complex. Cells were cultured for 4–6 hours, after which the culture medium was replaced with fresh complete medium, and incubation continued for 24–48 hours before subsequent experiments. Transfection efficiency was verified by qRT-PCR.

### 2.11 Cell proliferation and colony formation assay

The siRNA-transfected OVCAR3 and A2780 cells were fully digested, seeded into 96-well culture plates at 5000 cells per well in triplicate, and incubated at 37 °C for a total of four days. Absorbance was determined using the Cell Counting Kit-8 (CCK8) as follows: 10 μL of CCK-8 reagent was added to each well at the same time each day. The plates were then incubated for 2 hours in a normal culture environment protected from light. Finally, the optical density (OD) was measured at 450 nm according to the instructions. OVCAR3 and A2780 cells infected with SI-NCAPG2 or SI-NC were digested and seeded into 6-well plates at 1000 cells per well. They were cultured in RPMI-1640 medium supplemented with 10% FBS and 1% penicillin streptomycin solution in an atmosphere containing 5% CO_2_ at 37°C for 2 weeks, with the medium changed appropriately during the culture period. After a certain period, the medium was discarded, and the cells were washed twice with PBS. The cells were then fixed in methanol for 15 minutes, stained with crystal violet for 15 minutes, and subsequently manually photographed and counted.

### 2.12 Invasion, migration, and wound healing assay

Cell invasion and migration experiments were performed using 24-well Transwell chambers. A volume of 200 μL serum-free medium containing 6×10^4^ cells or 4×10^4^ cells was added to a Transwell chamber with and without Matrigel for invasion and migration tests, respectively. Then, 600 μL of medium containing 10% FBS was added to the lower chamber. Then, cells were cultivated for 48 hours. The invaded and migrated cells were fixed in methanol for 15 minutes and stained with crystalline violet for 15 minutes. Then, cells were photographed and counted using an imaging microscope. OVCAR3 and A2780 cells with NCAPG2-knockdown and corresponding normal control cells were seeded in 6-well plates and cultured until the cell monolayer reached confluence. Then the cells were scraped in a straight line with a sterile 200 μL pipette tip, and images were taken immediately at 0 hours. After removing cell debris by washing with PBS, the cells were incubated for 48 hours. The migration of the cells at 0 hours and 48 hours was observed under the microscope, and the relative mobility was calculated by analyzing the captured images.

### 2.13 Western blot

Cells were lysed in RIPA lysis buffer for 30 minutes, and the lysates were centrifuged at 12,000 g for 10 minutes. The supernatant fraction was collected for Wb analysis. Briefly, 60 μg of protein sample was separated on 10% SDS-polyacrylamide gels and transferred to polyvinylidene fluoride membranes. The membranes were blocked with 5% non-fat milk for 2 hours at room temperature, followed by overnight incubation at 4°C with the appropriate primary antibodies (E-cadherin, 1:1000; N-cadherin, 1:1000; Vimentin, 1:1000; GAPDH, 1:1000) (Abmart, Shanghai, China). Subsequently, membranes were incubated with HRP-conjugated anti-rabbit IgG or anti-mouse IgG secondary antibodies (1:20000) for 1 hour at room temperature (Proteintech Group, Wuhan, China). The membranes were then treated with an enhanced chemiluminescence kit (Meilunbio, Dalian, China). Protein expression levels were assessed using Image J software version.1.8.0 (National Institutes of Health, Bethesda, MD, USA). GAPDH was used as a loading control, and protein expression levels were normalized to GAPDH for statistical analysis.

### 2.14 Clinical specimens

A total of 15 normal ovarian tissues and 30 EOC tissues were collected from patients who were diagnosed with EOC and underwent surgical treatment at The Third Affiliated Hospital of Zhengzhou University between January 2016 and September 2022. Relevant clinical information and other data were collected through medical records. The inclusion criteria were as follows: a. complete clinical information was available; b. all patients were informed of the associated risks; c. all patients provided written informed consent; d. all patients agreed to provide relevant follow-up information. Furthermore, the exclusion criteria: a. patients who received adjuvant therapy or other treatments before surgery; b. patients who had other systemic malignancies. The study was approved by the Ethics Committee of The Third Affiliated Hospital of Zhengzhou University (2023-172-01). All procedures in our research were conducted in accordance with the Helsinki Declaration.

### 2.16 Statistical analysis

The data types used in this study mainly consist of count and vst data. We used count data for different gene analyses and vst data for the downstream analysis of NCAPG2. For the correlation of NCAPG2 with other genes, we performed Pearson correlation analysis. Statistical analysis and charting were conducted using GraphPad Prism 8.0 software (GraphPad Prism, San Diego, CA, USA) and SPSS 26.0 software (SPSS Inc., Chicago, IL, USA). The final data were presented as mean ± SD, and the results of the CCK-8 assay were analyzed by two-way ANOVA. For data that did not follow a normal distribution, two-group comparisons were made using the nonparametric Mann-Whitney U test. Error bars in the figures indicate quartiles, while error bars in the full-text statistical plots represent SD. Experiments were independently repeated 2–3 times. The significance of differences in NCAPG2 expression between different EOC cells, as well as differences between SI-NCAPG2 and SI-NC groups in each experiment, were determined using the unpaired two-tailed Student’s t-test. For correlation analyses, Pearson correlation analysis was used for normally distributed data, and spearman correlation was used for non-normal data. *P*-values for each analysis are marked on the figures. Differences were considered statistically significant at *P* < 0.05 (*P < 0.05; **P < 0.01; ***P < 0.001; ****P < 0.0001).

## 3 Result

### 3.1 Study design

Firstly, we downloaded the clinical RNA expression data and OC data from TCGA and GTEx through the UCSC-Xena database and performed data screening. Next, we analyzed the differential expression of NCAPG2 in EOC tissues compared with normal ovarian tissues. We also assessed the quality of the data samples and the homogeneity within the sample groups. Furthermore, we analyzed the genetic differences between normal and tumor tissues, and functionally enriched the DEGs. More importantly, we investigated the mechanism of NCAPG2 in EOC using bioinformatics approaches, including GSEA, the relationship between NCAPG2 expression and histological grading and staging, and the identification of genes most strongly correlated with NCAPG2 based on TCGA-GTEx data. In addition, we analyzed the role of NCAPG2 in the tumor immune microenvironment and performed drug sensitivity analysis of commonly used treatments for OC. To further explore how NCAPG2 contributes to the development of EOC, we conducted enrichment analysis and performed *in vitro* functional experiments to study the role of NCAPG2 in EOC cells.

### 3.2 NCAPG2 is highly expressed in EOC in bioinformatics

**Figure 3:**
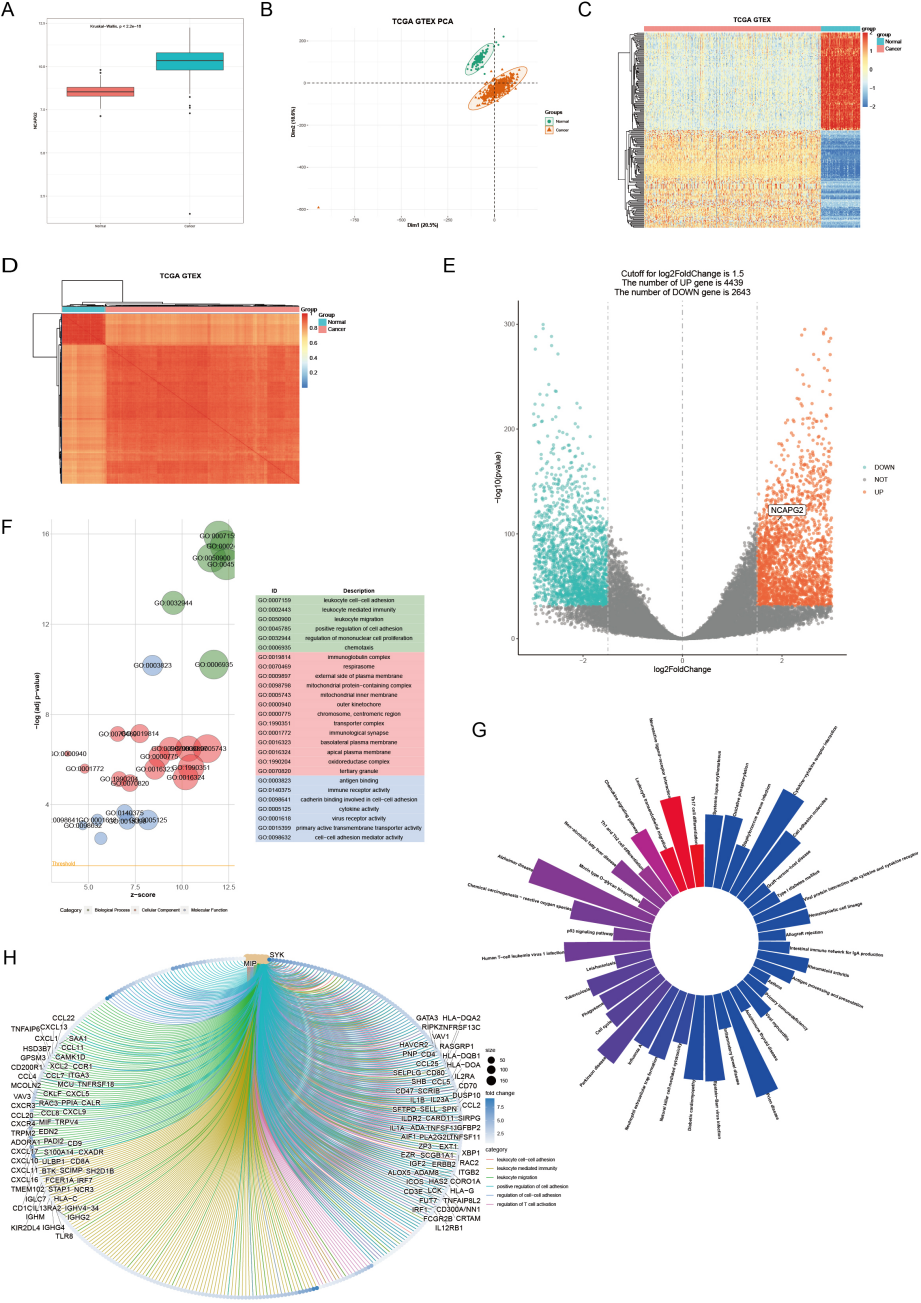
**(A)** Gene expression differences between 397 EOC tissues and 88 normal ovarian tissues in the TCGA database combined with the GTEx database. **(B)** PCA plot of normal and tumor tissues. **(C)** Heatmap of expression levels of the top 1000 differentially expressed genes (DEGs) in normal tissues versus tumor tissues (blue bar represents normal tissues and red represents tumor tissues). **(D)** Heatmap of correlation between normal and tumor tissue expression (blue bar represents normal tissues and red represents tumor tissues; the darker the color, the higher or lower the expression). **(E)** Volcano plot of DESeq2 analysis of differences between normal and tumor tissues (cutoff is 1.5; p = 1.0 × 10^−^³²); **(F)** GO enrichment analysis of the DEGs between normal and tumor tissues. **(G)** KEGG enrichment analysis of the differentially expression genes between normal and tumor tissues. **(H)** Visualization of gene correlations within the pathways of the GO enrichment analysis.

Due to the limited normal tissue sample size in the TCGA database, expression profile data from normal tissues were supplemented from the GTEx database. The results of the analysis show that NCAPG2 was highly expressed in EOC tissues (**Figure 3A**). Before the differential gene analysis, we downloaded count-format data processed by RSEM from the UCSC database, which we then transformed into vst format to perform principal component analysis (PCA) comparing normal and tumor tissues (**Figure 3B**). The PCA results showed a clear separation between EOC and normal tissues in the dataset. The variance of gene expression across all tissues was calculated and sorted. The top 1000 genes with the highest variance were selected, and a heat map comparing normal and tumor tissues was generated (**Figure 3C**). Correlation analysis between tissue samples showed clustering according to tissue type (**Figure 3D**). **Figures 3B–D** demonstrate good sample quality and homogeneity within sample groups. Therefore, the data are reliable and suitable for further analysis. Differential expression analysis between normal and tumor tissues was performed using DESeq2. In total, 2643 genes were down-regulated and 4439 genes were up-regulated in EOC tissues, including NCAPG2, indicating that NCAPG2 is significantly up-regulated in EOC (**Figure 3E**). GO enrichment analysis was performed for DEGs and revealed enrichment in gene sets related to leukocyte cell-cell adhesion, leukocyte-mediated immunity, and leukocyte migration (**Figure 3F**). KEGG enrichment analysis of the DEGs showed significant enrichment in the systemic lupus erythematosus, oxidative phosphorylation, and staphylococcus aureus infection pathways (**Figure 3G**). We also performed circular visualization of gene correlations within the enriched GO pathways (**Figure 3H**).

### 3.3 Analysis of the mechanism of action of NCAPG2 in bioinformatics

**Figure 4:**
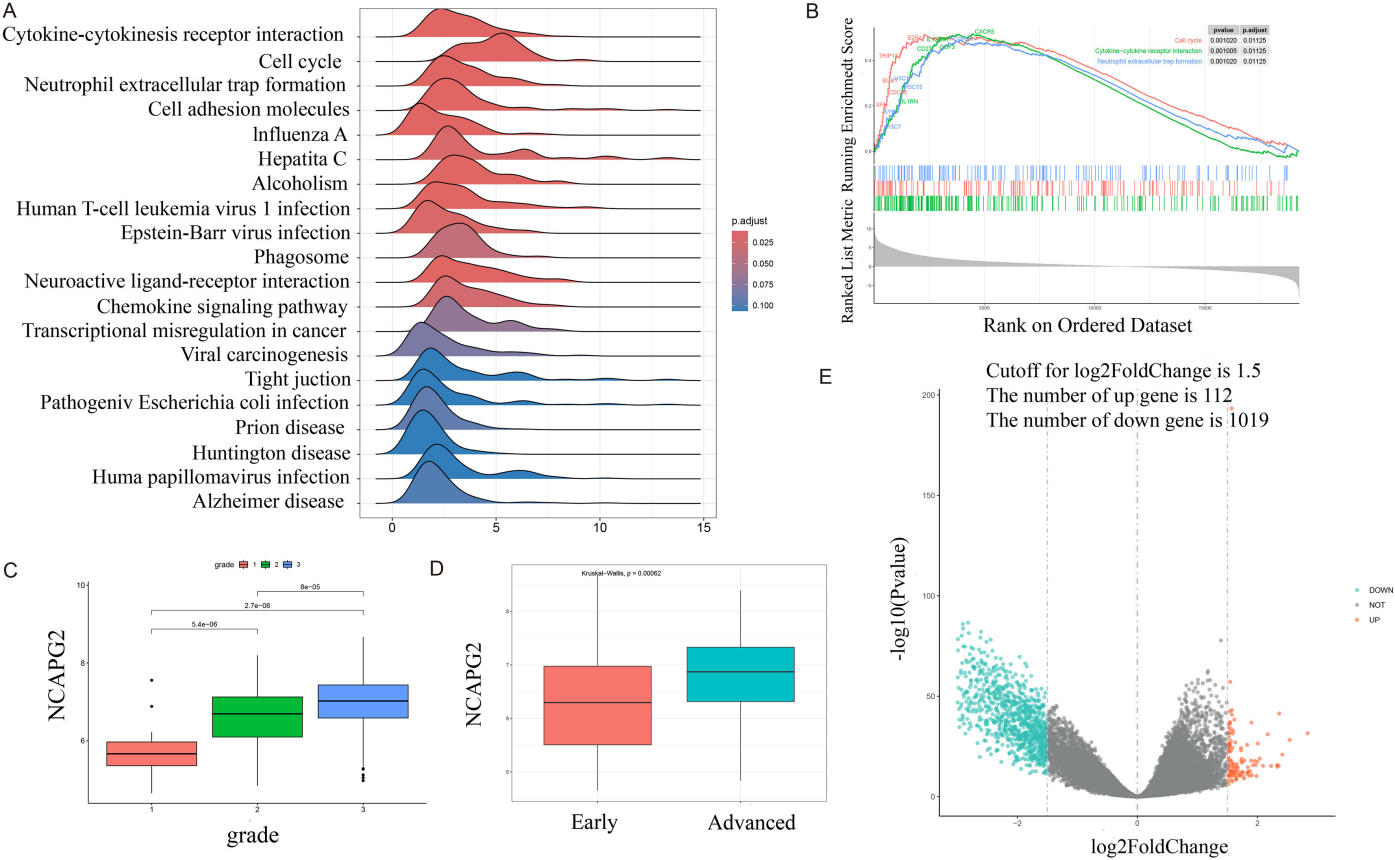
**(A)** GSEA Ridgeline Plot of all genes according to the results of differential analysis between normal tissues and tumors. **(B)** The top 3 pathways identified by GSEA of all genes according to the results of differential analysis between normal tissues and tumors. **(C)** Differential expression of NCAPG2 at different grades (GSE9891). **(D)** Differential expression of NCAPG2 at different stages (Early stages: I, II; Advanced stages: III, IV) (GSE9891). **(E)** Volcano plot of DEGs after grouping samples by high and low expression of NCAPG2 in TCGA-GTEx samples.

**Figure 5:**
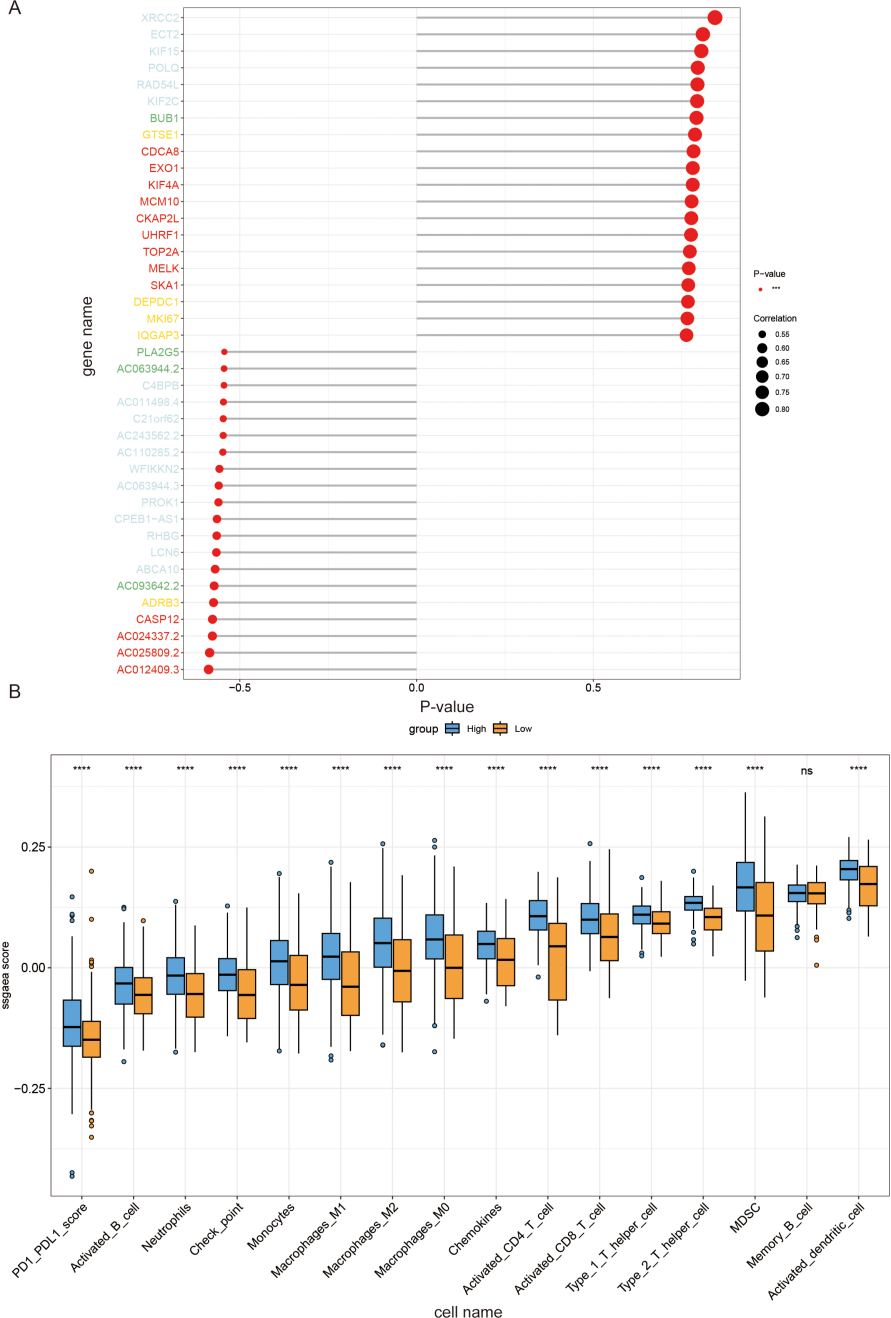
(A) Visualization of genes with stronger positive and negative correlations with NCAPG2. (B) Correlation between 16 immune cell infiltration scores and NCAPG2 gene expression in EOC patients. The 16 immune cells analyzed include: PD-1/PD-L1 score, Activated B cells, Neutrophils, Checkpoint, Monocytes, M1 Macrophages, M2 Macrophages, M0 Macrophages, hemokines, Activated CD4 T cell, Activated CD8 T cell, Type 1 T helper cells, Type 2 T helper cells, Myeloid-Derived Suppressor Cells (MDSC), Memory B cells, and Activated dendritic cells. (ns P≥0.05; ****P < 0.0001).

**Figure 6:**
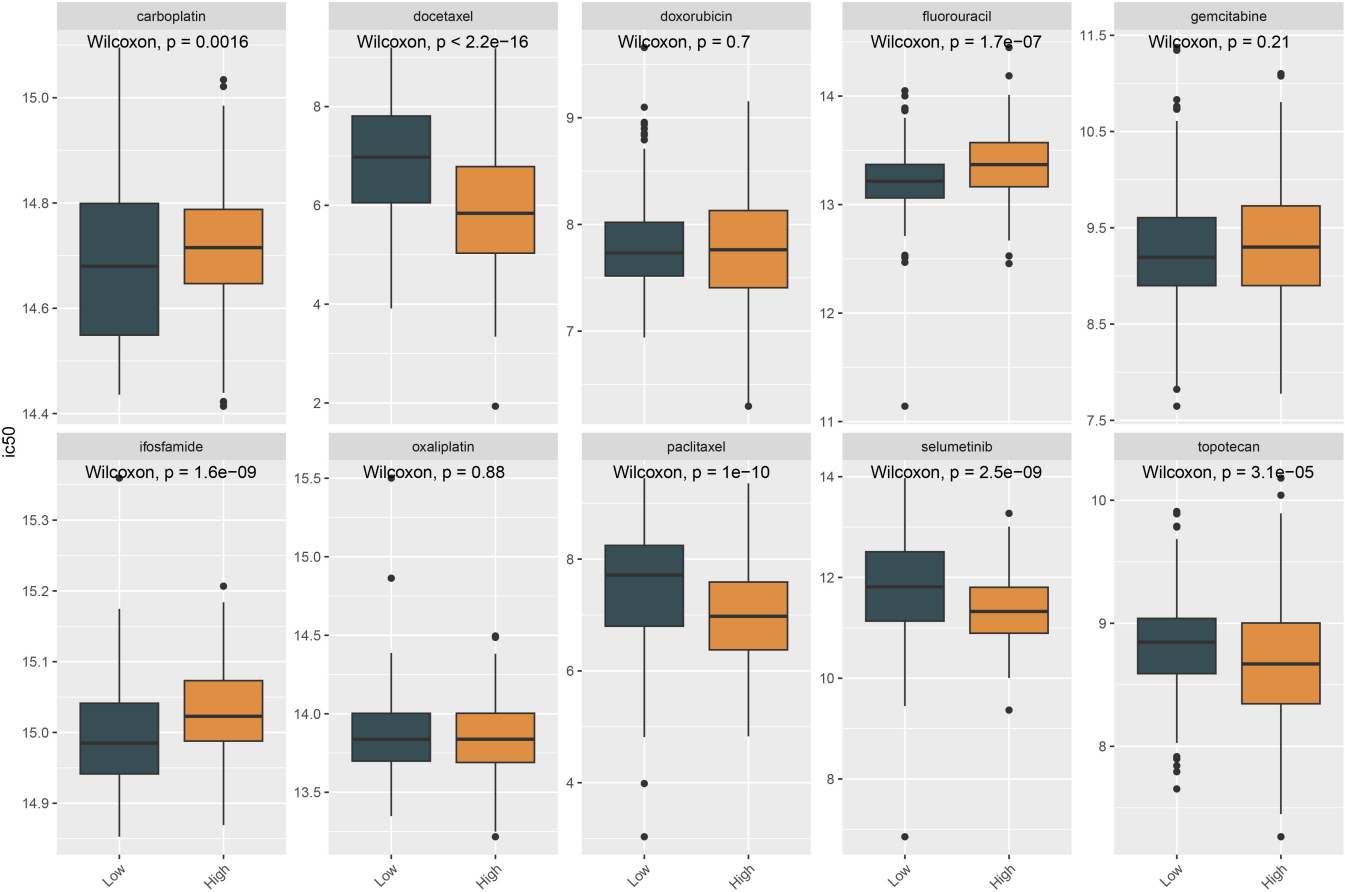
NCAPG2-related drug susceptibility analysis using TCGA-GTEx data. The group with higher NCAPG2 expression exhibited a higher drug half maximal inhibitory concentration (IC50), indicating that a higher drug concentration was required to inhibit the targeted biological process, suggesting that cells with elevated NCAPG2 expression were more resistant to the drug. Conversely, in some cases, the group with higher NCAPG2 expression showed a lower drug IC50, indicating increased sensitivity to the drug.

Differential analysis between normal tissues and tumors was performed, and GSEA ridge plots were generated for all genes (**Figure 4A**). The top three pathways, which may be related to the cell cycle, cytokine-cytokine receptor interaction, and neutrophil extracellular trap formation, were plotted separately (**Figure 4B**). NCAPG2 expression was correlated with histological grade and stage; higher histological grades or later stages were associated with increased expression (**Figures 4C, D**). A volcano plot was generated after grouping NCAPG2 gene expression using TCGA-GTEx data, followed by differential analysis between high and low expression samples using DESeq2 (**Figure 4E**). Using TCGA-GTEx data, the top 20 positively and top 20 negatively correlated genes with NCAPG2 were analyzed. The strongest positive correlations were with XRCC2, ECT2, and KIF15, while the strongest negative correlation was with AC012409.3 (**Figure 5A**). Samples were divided according to the maximum cut-off value for ssGSEA immune infiltration analysis. The results showed that high NCAPG2 expression may be associated with PD-1/PD-L1 score, activated B cells, neutrophils, immune checkpoints, monocytes, M1 macrophages, M2 macrophages, M0 macrophages, chemokines, activated CD4 T cells, activated CD8 T cells, type 1 T helper cells, type 2 T helper cells, myeloid-derived suppressor cells (MDSC), and activated dendritic cells; however, there was no correlation with memory B cells (**Figure 5B**). To further explore the clinical value of NCAPG2, we conducted drug sensitivity analysis using OncoPredict based on immune infiltration grouping. We selected drugs commonly used in the clinical treatment of OC and added the targeted drug selumetinib. Drug sensitivity analysis was performed on the TCGA-GTEx data. The results suggest that NCAPG2 expression may be associated with sensitive to docetaxel, paclitaxel, selumetinib, and topotecan, and resistance to carboplatin, fluorouracil, and ifosfamide (**Figure 6**).

### 3.4 NCAPG2 expression is correlated with DNA damage repair gene signatures

**Figure 7:**
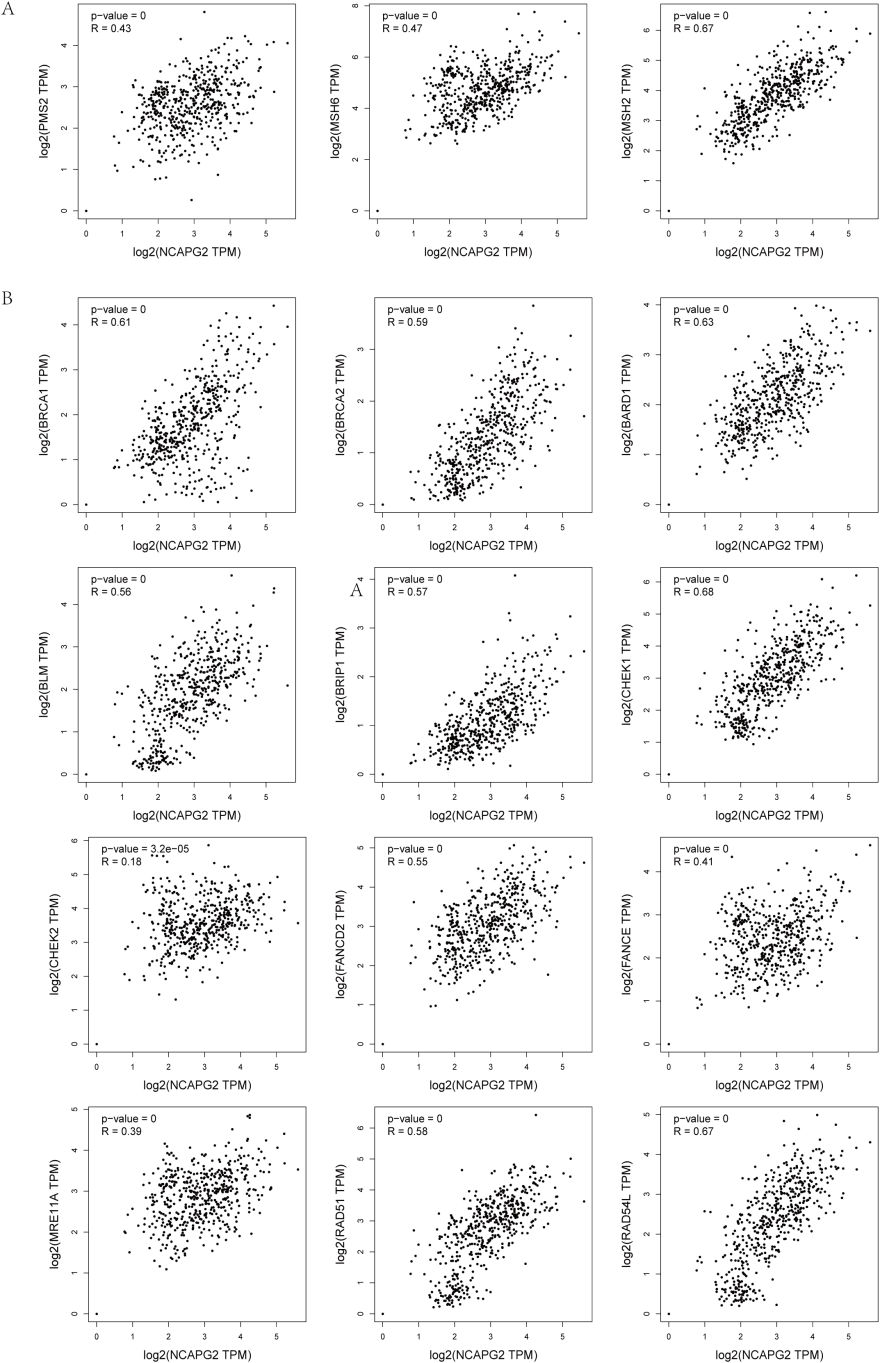
**(A)** Correlation analysis between NCAPG2 and the key genes of MMR. **(B)** Correlation analysis between NCAPG2 and the key genes of HRR.

To explore the potential impact of NCAPG2 on genome stability, we investigated its relationship with DNA damage response and repair pathways. We used the GEPIA2 database to analyze the correlation between NCAPG2 and key genes expression in the MMR and HRR pathways. Importantly, NCAPG2 mRNA levels showed significant positive correlations with the majority of these DDR genes. Specifically, NCAPG2 was positively correlated with the MMR genes PMS2 (*R* = 0.43, *P* < 0.001), MSH6 (*R* = 0.47, *P* < 0.001), and MSH2 (*R* = 0.67, *P* < 0.001) (**Figure 7A**). Furthermore, strong positive correlations were observed with critical HRR genes, including BRCA1 (*R* = 0.61, *P* < 0.001), BRCA2 (*R* = 0.59, *P* < 0.001), BARD1 (*R* = 0.63, *P* < 0.001), BLM (*R* = 0.56, *P* < 0.001), BRIP1 (*R* = 0.57, *P* < 0.001), CHEK1 (*R* = 0.68, *P* < 0.001), CHEK2 (*R* = 0.18, *P* < 0.001), FANCD2 (*R* = 0.55, *P* < 0.001), FANCE (*R* = 0.41, *P* < 0.001), MRE11A (*R* = 0.39, *P* < 0.001), RAD51 (*R* = 0.58, *P* < 0.001), and RAD54L (*R* = 0.67, *P* < 0.001) (**Figure 7B**).

### 3.5 NCAPG2 expression is correlated with chemotherapy resistance-related genes

**Figure 8:**
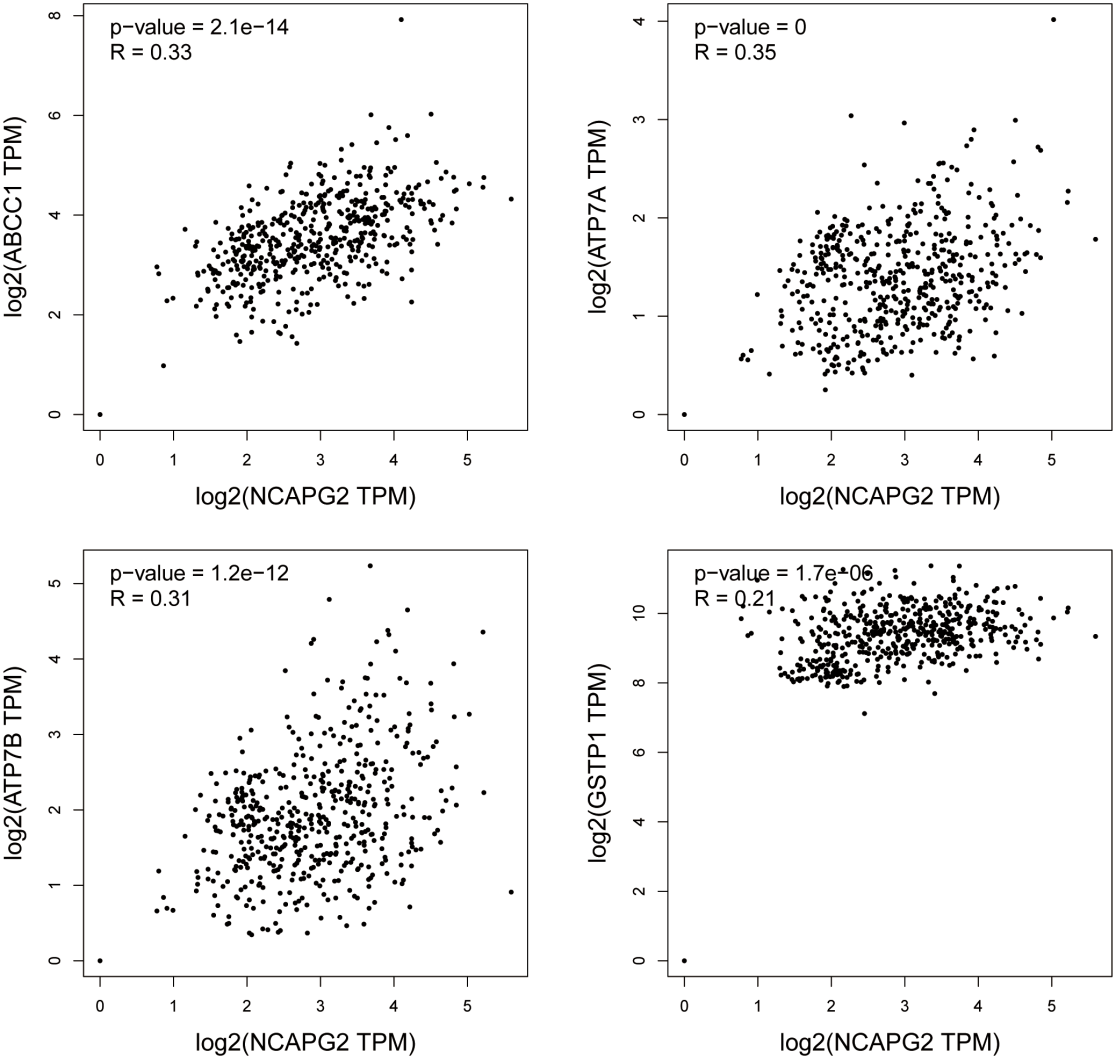
Correlation analysis between NCAPG2 and chemotherapy resistance-related genes.

To directly investigate the potential role of NCAPG2 in chemoresistance, we analyzed its correlation with genes associated with chemotherapy resistance. Using the GEPIA2 platform, our results showed that NCAPG2 mRNA expression was significantly and positively correlated with the expression of four major chemotherapy resistance-related genes: ABCC1 (MRP1), *R* = 0.33, *P* < 0.001; ATP7A, *R* = 0.35, *P* < 0.001; ATP7B, *R* = 0.31, *P* < 0.001; GSTP1, *R* = 0.21, *P* < 0.05; (**Figure 8**).

### 3.6 Gene enrichment analysis of NCAPG2 in bioinformatics

**Figure 9:**
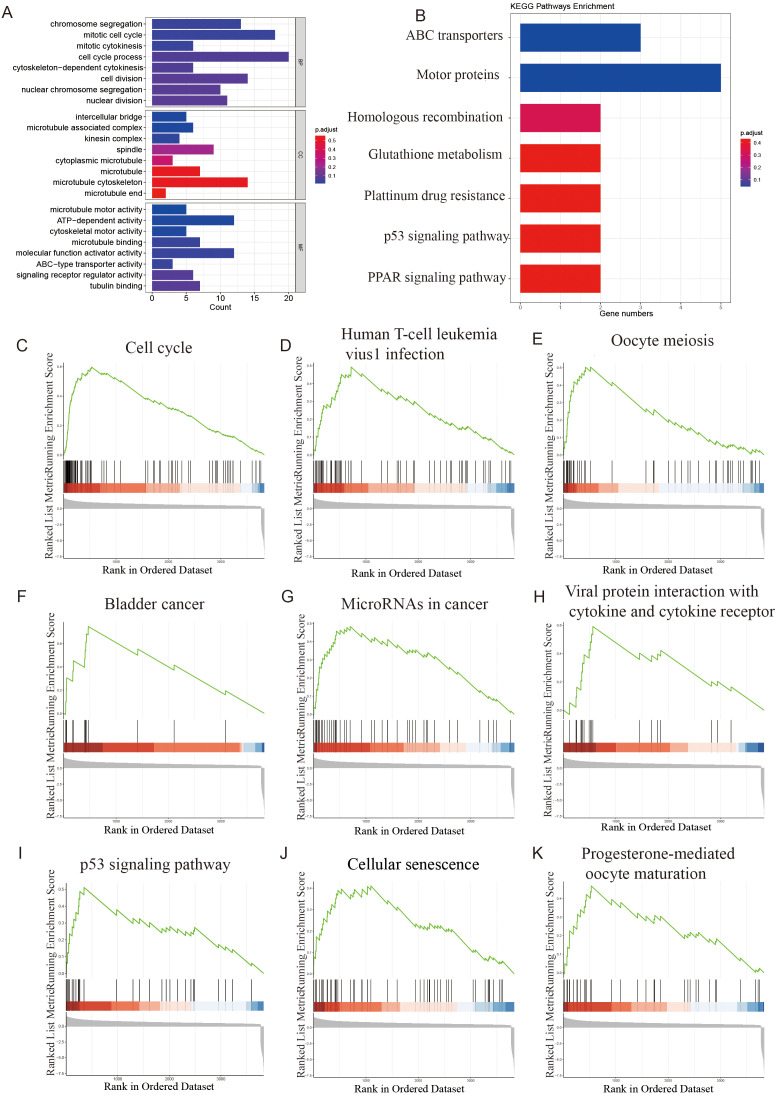
Enrichment analysis of NCAPG2 in EOC (p<0.05). **(A)** GO enrichment analysis of NCAPG2 in EOC. **(B)** KEGG enrichment analysis of NCAPG2 in EOC. **(C-K)** GSEA enrichment analysis of NCAPG2 in EOC (the top 9 pathways).

We selected genes with an absolute correlation coefficient greater than 0.5 for NCAPG2 for enrichment analysis. We further explored the potential functional pathways of these genes using the R software, clusterProfiler package. GO functional enrichment analysis showed that NCAPG2 was mainly associated with chromosome segregation, intercellular bridges, microtubule motor activity, and other related pathways (**Figure 9A**). The results of the KEGG analysis indicated that NCAPG2 was closely related to ABC transporters, motor proteins, and additional pathways (**Figure 9B**). To further investigate, we performed GSEA on genes differentially expressed in relation to NCAPG2. We found that the pathways significantly enriched included the cell cycle, human T-cell leukemia virus 1 infection, oocyte meiosis, bladder cancer, microRNAs in cancer, viral protein interaction with cytokine and cytokine receptor, cellular senescence, progesterone-mediated oocyte maturation, and the tumor-related P53 signaling pathway (**Figures 9C–K**). These results demonstrated that high expression of NCAPG2 was associated with multiple oncogenic pathways in EOC.

### 3.7 Expression of NCAPG2 in EOC cells and its effect on the cell proliferative, migration, and invasion capacity *in vitro*

**Figure 10:**
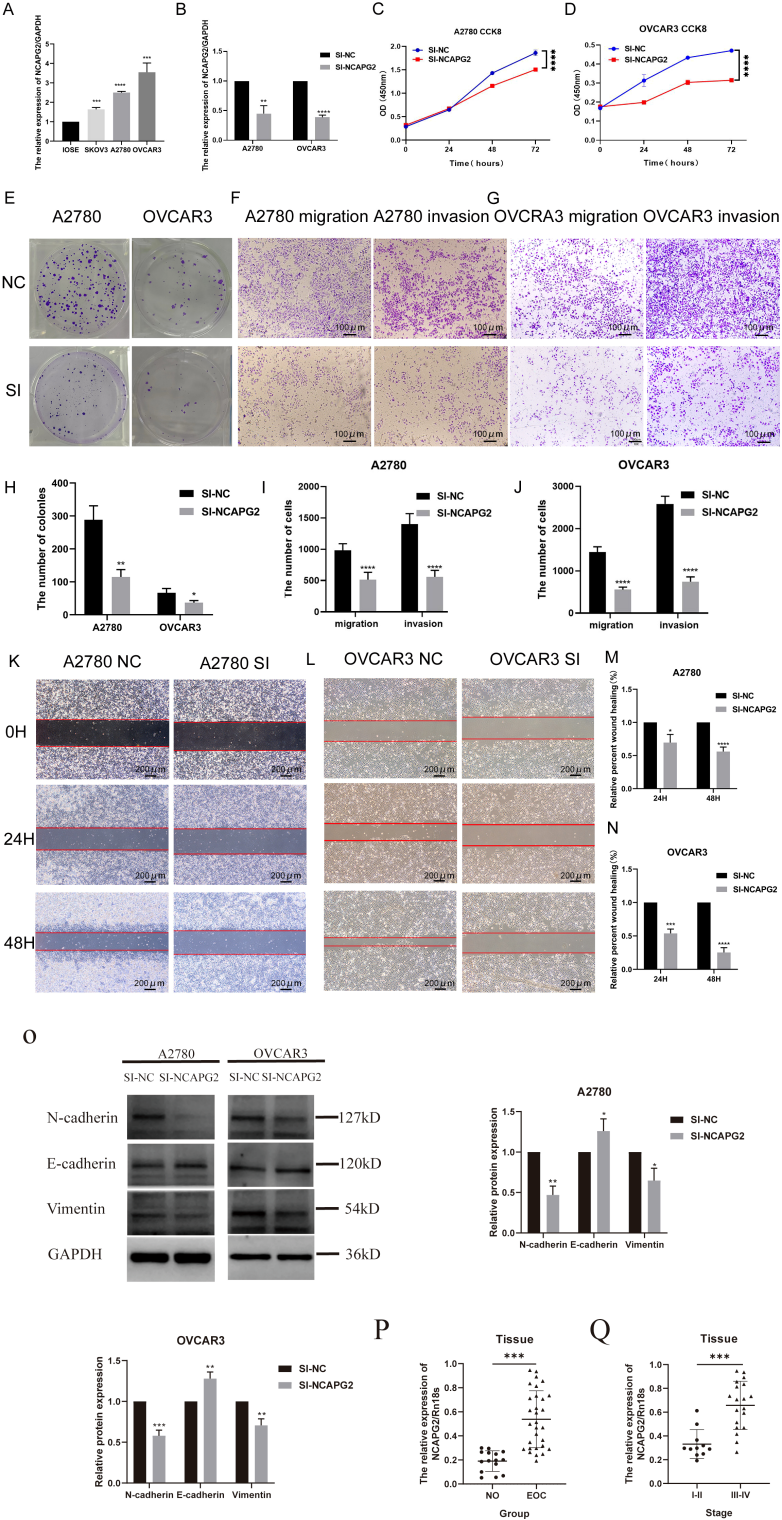
Vitro proliferation and migration capacity of EOC cells in the NCAPG2 knockdown and control groups. **(A)** qRT-PCR was used to detect the difference in gene expression of NCAPG2 at the cellular level between various EOC cells and normal ovarian cells. **(B)** The relative expression of NCAPG2 mRNA in A2780 and OVCAR3 cells in NCAPG2 knockdown and control group was verified by qRT-PCR. **(C, D)** CCK8 cell proliferation assay in SI-NCAPG2 A2780 cells and SI-NCAPG2 OVCAR3 cells. **(E, H)** Colony formation assay photos and bar graphs of colony-forming cell counts in SI-NCAPG2 A2780 cells and SI-NCAPG2 OVCAR3 cells. **(F, G, I, J)** Transwell migration and invasion assay photos and bar graphs of migrated and invaded cell counts in SI-NCAPG2 A2780 cells and SI-NCAPG2 OVCAR3 cells. **(K, M)** Wound-healing assay bar graphs and photos of healing ratios in SI-NCAPG2 A2780 cells; **(L, N)** Wound-healing assay bar graphs and photos of healing ratios in SI-NCAPG2 OVCAR3 cells. (*P < 0.05; **P < 0.01; ***P < 0.001; ****P < 0.0001). **(O)** Representative images and quantitative analysis of A2780 and OVCAR3 cells transfected with SI-NC or SI-NCAPG2 siRNA to detect N-cadherin, E-cadherin, and Vimentin expression. **(P)** The differential expression of NCAPG2 mRNA in epithelial ovarian cancer (EOC, n=30) versus normal ovary (NO, n=15). **(Q)** The differential expression of NCAPG2 mRNA in EOC tissues from patients with different FIGO stages (FIGO: International Federation of Gynecology and Obstetrics; I-II, n=11; III-IV, n=19). (*P < 0.05; **P < 0.01; ***P < 0.001; ****P < 0.0001).

According to the qRT-PCR results (**Figure 10A**), due to the relatively high expression of NCAPG2 in both A2780 (*P* < 0.0001) and OVCAR3 (*P* = 0.0008) cells, these two cell lines were selected for transfection. Subsequent cell function validation experiments were performed. We transfected NCAPG2 siRNA into A2780 and OVCAR3 cells qRT-PCR experiments were used to verify transfection efficiency (**Figure 10B**); the knockdown efficiency reached approximately 55% and 61%, respectively, in A2780 (*P* = 0.0021) and OVCAR3 (*P* < 0.0001) cells. The results of CCK-8 assays (**Figures 10C, D**) and colony formation assays (**Figures 10E, H**) showed that the knockdown of NCAPG2 inhibited the proliferation of EOC cells in both A2780 (*P* < 0.0001) and OVCAR3 (*P* < 0.0001) cells compared to the control group. Similar results were obtained from the colony formation assay (A2780 (*P* = 0.0033) and OVCAR3 (*P* = 0.0244)). Subsequently, the Transwell assay demonstrated that silencing NCAPG2 significantly impaired the migration ability of A2780 cells (*P* < 0.0001). In the invasion experiment, we also observed that knocking down NCAPG2 reduced the number of cells invading through Matrigel, with the difference being statistically significant (*P* < 0.0001) (**Figures 10F, I**). These results were similarly validated in OVCAR3 cells (migration (*P* < 0.0001), invasion (*P* < 0.0001)) (**Figures 10G, J**).

To investigate whether NCAPG2 affects the migration rate of A2780 and OVCAR3 cells and to verify its effect on their migration ability by various methods, we performed NCAPG2 knockdown and assessed changes in migration ability using a wound healing assay. As shown in **Figures 10K–N**, the relative percent wound healing (%) in A2780 cells was decreased by 30% at 24 hours (*P* < 0.05) and 44% at 48 hours (*P* < 0.0001) after NCAPG2 knockdown. Moreover, in OVCAR3 cells, the relative percent wound healing (%) was decreased by 46% at 24 hours (*P* < 0.001) and 75% at 48 hours (*P* < 0.0001) in the SI-NCAPG2 group. Through the above experimental analyses, our study confirmed that silencing NCAPG2 attenuated the proliferation, migration, and invasion capacities of EOC cells *in vitro*. To further investigate the mechanism underlying NCAPG2-mediated promotion of migration and invasion, we assessed the effect of NCAPG2 silencing on the expression of epithelial-mesenchymal transition (EMT) markers. Wb analysis revealed that silencing NCAPG2 significantly upregulated the protein level of the epithelial marker E-cadherin in both A2780 (*P* < 0.05) and OVCAR3 (*P* < 0.01) cells (**Figure 10O**). Conversely, the expression of key mesenchymal markers, including N-cadherin and Vimentin, was markedly downregulated after NCAPG2 silencing in both A2780 (*P* < 0.05) and OVCAR3 (*P* < 0.05) cells (**Figure 10O**). These alterations in EMT marker expression were consistent in both cell lines. These results indicate that NCAPG2 is a critical regulator of EMT, and its silencing can effectively inhibit the EMT process, shifting cells towards a more epithelial and less invasive state. The expression of NCAPG2 mRNA was significantly higher in EOC tissues compared to normal ovarian tissues (*P* < 0.05, **Figure 10P**), based on qRT-PCR results of ovarian tissues obtained from The Third Affiliated Hospital of Zhengzhou University. We further investigated the difference in NCAPG2 mRNA expression between in early (stage I-II) and advanced (stage III-IV) EOC tissues. The results showed that NCAPG2 mRNA levels were significantly elevated in advanced EOC tissues compared with early stages (*P* < 0.001, **Figure 10Q**).

## 4 Discussion

OC, the second most common gynecological tumor, affects women of all ages, with surgery and chemotherapy as primary treatments (32). Despite advancements, the five-year survival rate is low (32). The progression mechanism of OC is not fully understood, but the immunosuppressive tumor microenvironment significantly impacts it (33). Finding effective targets is crucial for improving survival rates. This study investigates how NCAPG2 promotes OC progression and chemotherapy resistance. NCAPG2, a subunit of the collectin II complex, regulates mitotic chromosome condensation and is crucial for protein sorting (34, 35). It is highly expressed in various cancers (15–18), but its role in EOC is unclear. Bioinformatics analysis and cytological experiment reveal NCAPG2’s expression profile and functional role in EOC. Differential analysis links the progression of EOC to leukocyte adhesion, immunity, migration, lupus, oxidative phosphorylation and Staphylococcus aureus infection, guiding future research on the mechanism of EOC. Furthermore, the expression of NCAPG2 is related to the histological grade and stage of EOC, suggesting that it is involved in the occurrence and progression of the disease. The development of tumors is related to genetic changes and the tumor microenvironment (36), among which immune cells (37) play a crucial role.

Previous reports have shown that the expression of NCAPG2 is closely related to the level of immune cell infiltration during the progression of lung adenocarcinoma (17, 38). The results of this study show that NCAPG2 is positively correlated with the infiltration of various immune cells other than memory B cells in EOC, suggesting that NCAPG2 may be involved in the regulation of the tumor immune microenvironment in EOC. Previous studies have shown that NCAPG2 is significantly correlated with immune cell infiltration, immune checkpoint genes, tumor mutational burden (TMB), microsatellite instability (MSI), and other related factors (39). Thus, NCAPG2 may become a new direction for EOC immunotherapy. Previous studies have reported that NCAPG2 is a key gene for drug resistance in various tumors (40), but the relationship between NCAPG2 and chemotherapy resistance in EOC remains unclear. Accordingly, in this study, we analyzed the relationship between the expression of the NCAPG2 gene and drug sensitivity. The results suggest that high expression of NCAPG2 is associated with increased resistance to carboplatin, fluorouracil, and ifosfamide. The possible mechanism is that high expression of NCAPG2 can promote the migration of EOC cells; induce an immunosuppressive tumor microenvironment; and facilitate cellular DNA repair, thereby contributing to chemotherapy resistance. These findings are consistent with the results reported in previous articles (41, 42).

NCAPG2 exhibits characteristics of an oncogene, yet its expression positively correlates with DNA repair genes. This suggests a more nuanced role for NCAPG2 in cancer biology beyond simply promoting proliferation. By ensuring high expression of key DNA repair machinery, NCAPG2 enables cancer cells to efficiently repair the DNA damage that is inherently associated with their high replication rates and metabolically stressful microenvironment. This allows them to avoid the two extremes: excessive damage leading to cell death, and perfect stability that might limit adaptive evolution (43). Simultaneously, its role in enhancing repair capacity provides a direct mechanism for resisting DNA-damaging therapies, such as platinum drugs, radiation (44). Cells with high NCAPG2 can better repair therapy-induced damage, leading to treatment failure. As shown in our bioinformatics analysis, the expression of NCAPG2 was positively correlated with some resistance-related genes. This therapeutic vulnerability could be exploited. Targeting NCAPG2, especially in combination with DNA-damaging agents, could induce synthetic lethality to overwhelm the cancer cell’s repair systems, offering a promising novel therapeutic strategy for EOC.

NCAPG2 overexpression promotes cell proliferation and migration in NSCLC cells (17).

However, no study has reported the function of NCAPG2 in EOC cell lines. To verify the oncogenic effect of NCAPG2 in EOC, our *in vitro* assays showed that NCAPG2 was highly expressed in EOC cells and that knockdown of NCAPG2 significantly reduced the proliferation and migration abilities of EOC cells. To further investigate the mechanism underlying NCAPG2-mediated promotion of migration and invasion, we investigated and found that silencing NCAPG2 significantly upregulates the expression of epithelial markers E-cadherin and downregulates the expression of mesenchymal markers N-cadherin and Vimentin, indicating that NCAPG2 is a key driver of the EMT process. The increase in E-cadherin, a key mediator of cell-cell adhesion, is consistent with the reduction in cellular motility (45). Additionally, the decrease in N-cadherin and Vimentin, which promote cell detachment and migration (45), directly accounts for the impaired invasive capabilities observed upon NCAPG2 knockdown. Thus, NCAPG2 promotes EOC cell invasion and migration by driving the EMT program. This finding offers a mechanistic explanation for the observed suppression of migration and invasion upon NCAPG2 silencing. Consequently, NCAPG2 is able to promote EOC progression by facilitating the metastasis of EOC cells. Our findings position NCAPG2 as a novel upstream regulator of EMT in EOC. We further confirmed in clinical tissue specimens that NCAPG2 is highly expressed in total EOC tissues. Moreover, its expression level significantly increases in advanced EOC tissues, indicating that NCAPG2 plays a key role in the progression of EOC. *In vitro* assays have confirmed the role of NCAPG2 in promoting tumor development in EOC; however, its mechanism of action requires further investigation.

This study examines NCAPG2’s role in EOC progression and proposes potential biomarkers. However, it faces limitations, such as reliance on public data rather than clinical studies. Future research should involve larger cohorts and *in vivo* experiments to better understand NCAPG2’s functions and its regulatory role in EOC, including its upstream and downstream interactions.

## 5 Conclusion

High expression of NCAPG2 in EOC can promote the progression of EOC and is closely related to immune response, DNA damage repair and chemotherapy resistance. Therefore, NCAPG2 may serve as an immunotherapy target for EOC.

## Data Availability

The datasets presented in this study can be found in online repositories. The names of the repository/repositories and accession number(s) can be found in the article/**Supplementary Material**.
