# Supplementary material for: Bioinformatics and experimental analysis revealed the cancer-promoting role of NCAPG2 in epithelial ovarian cancer
Source: Front Oncol. 2026 Mar 13;16:1574236. doi: 10.3389/fonc.2026.1574236 (PMC13021424; doi:10.3389/fonc.2026.1574236)
Supplement: Supplementary file 1 [file Table1.docx]

Summary descriptives table by groups of `group'

|  | **High** | **Low** | **p.overall** |
| --- | --- | --- | --- |
|  | ***N=243*** | ***N=242*** |  |
| Activated_B_cell | -0.03 (0.05) | -0.05 (0.05) | <0.001 |
| Activated_CD4_T_cell | 0.11 (0.04) | 0.02 (0.09) | <0.001 |
| Activated_CD8_T_cell | 0.10 (0.05) | 0.07 (0.06) | <0.001 |
| Activated_dendritic_cell | 0.20 (0.03) | 0.17 (0.05) | <0.001 |
| aDCs | 0.15 (0.08) | 0.06 (0.11) | <0.001 |
| Angiogenesis | 0.10 (0.05) | 0.13 (0.08) | <0.001 |
| Antigen_Processing_and_Presentation | 0.27 (0.03) | 0.24 (0.04) | <0.001 |
| Antimicrobials | 0.14 (0.03) | 0.11 (0.03) | <0.001 |
| APC_co_inhibition | 0.09 (0.07) | 0.04 (0.08) | <0.001 |
| APC_co_stimulation | -0.07 (0.05) | -0.10 (0.06) | <0.001 |
| APM1 | 0.46 (0.03) | 0.44 (0.04) | <0.001 |
| APM2 | 0.22 (0.10) | 0.14 (0.15) | <0.001 |
| B_Cell | 0.21 (0.02) | 0.19 (0.03) | <0.001 |
| B_Cell_60gene | 0.12 (0.16) | 0.06 (0.17) | <0.001 |
| B_Cell_cluster | -0.17 (0.08) | -0.20 (0.08) | <0.001 |
| B_cell_PCA_16704732 | 0.21 (0.02) | 0.19 (0.03) | <0.001 |
| B_cells | -0.09 (0.05) | -0.10 (0.05) | 0.027 |
| B_cells_ImSig | -0.12 (0.05) | -0.14 (0.05) | <0.001 |
| B_cells_memory | 0.00 (0.06) | -0.02 (0.06) | <0.001 |
| B_cells_naive | 0.01 (0.05) | -0.01 (0.05) | <0.001 |
| B_lineage_MCPcounter | -0.05 (0.08) | -0.07 (0.07) | 0.025 |
| Bcell_21978456 | 0.12 (0.19) | 0.06 (0.20) | 0.002 |
| Bcell_mg_IGJ | 0.19 (0.14) | 0.12 (0.16) | <0.001 |
| Bcell_receptors_score | 0.06 (0.05) | 0.02 (0.05) | <0.001 |
| BCRSignalingPathway | 0.10 (0.09) | 0.05 (0.10) | <0.001 |
| Buck14_score | 0.15 (0.03) | 0.15 (0.03) | 0.039 |
| CCR | 0.05 (0.04) | 0.01 (0.05) | <0.001 |
| CD103neg_mean_25446897 | 0.17 (0.07) | 0.14 (0.09) | <0.001 |
| CD103pos_mean_25446897 | -0.07 (0.04) | -0.09 (0.03) | <0.001 |
| CD56bright_natural_killer_cell | 0.19 (0.02) | 0.19 (0.02) | 0.023 |
| CD56dim_natural_killer_cell | 0.23 (0.02) | 0.21 (0.03) | <0.001 |
| CD68 | 0.36 (0.12) | 0.20 (0.22) | <0.001 |
| CD68_cluster | 0.08 (0.09) | 0.00 (0.12) | <0.001 |
| CD8 | -0.01 (0.06) | -0.05 (0.06) | <0.001 |
| CD8_CD68_ratio | 0.18 (0.10) | 0.07 (0.16) | <0.001 |
| CD8_cluster | -0.11 (0.09) | -0.16 (0.09) | <0.001 |
| CD8_PCA_16704732 | 0.00 (0.06) | -0.04 (0.07) | <0.001 |
| CD8_T_cells_MCPcounter | -0.15 (0.12) | -0.22 (0.14) | <0.001 |
| CD8._T_cells | -0.03 (0.12) | -0.08 (0.13) | <0.001 |
| CD8A | -0.03 (0.12) | -0.08 (0.13) | <0.001 |
| Central_memory_CD4_T_cell | 0.32 (0.02) | 0.30 (0.04) | <0.001 |
| Central_memory_CD8_T_cell | 0.20 (0.03) | 0.19 (0.04) | <0.001 |
| CHANG_CORE_SERUM_RESPONSE_UP | 0.28 (0.02) | 0.25 (0.04) | <0.001 |
| Check_point | -0.01 (0.05) | -0.05 (0.06) | <0.001 |
| Chemokine_Receptors | 0.05 (0.04) | 0.04 (0.04) | 0.105 |
| Chemokine12_score | 0.03 (0.12) | -0.06 (0.15) | <0.001 |
| Chemokines | 0.05 (0.04) | 0.01 (0.05) | <0.001 |
| CSF1_response | 0.18 (0.08) | 0.12 (0.09) | <0.001 |
| CSR_Activated_15701700 | 0.30 (0.01) | 0.30 (0.02) | <0.001 |
| CTLA4_data | -0.23 (0.12) | -0.28 (0.12) | <0.001 |
| Cytokine_Receptors | 0.08 (0.02) | 0.08 (0.02) | 0.605 |
| Cytokines | 0.01 (0.02) | 0.00 (0.03) | <0.001 |
| Cytolytic_activity | -0.09 (0.11) | -0.15 (0.12) | <0.001 |
| Cytotoxic_lymphocytes_MCPcounter | -0.14 (0.09) | -0.17 (0.09) | <0.001 |
| DAP12_data | 0.35 (0.11) | 0.22 (0.21) | <0.001 |
| DCs | -0.20 (0.08) | -0.25 (0.10) | <0.001 |
| Dendritic_cells_activated | 0.02 (0.07) | -0.03 (0.07) | <0.001 |
| Dendritic_cells_resting | 0.03 (0.08) | -0.03 (0.09) | <0.001 |
| Effector_memory_CD4_T_cell | 0.14 (0.02) | 0.12 (0.02) | <0.001 |
| Effector_memory_CD8_T_cell | 0.14 (0.05) | 0.14 (0.04) | 0.520 |
| Endothelial_cells_MCPcounter | 0.02 (0.04) | 0.04 (0.07) | <0.001 |
| Eosinophil | -0.03 (0.04) | -0.03 (0.04) | 0.979 |
| Eosinophils | -0.02 (0.06) | -0.05 (0.06) | <0.001 |
| Fibroblasts_MCPcounter | 0.35 (0.05) | 0.35 (0.06) | 0.983 |
| G_CD3E | -0.05 (0.11) | -0.10 (0.11) | <0.001 |
| G_CYTH4 | 0.06 (0.10) | -0.01 (0.12) | <0.001 |
| G_GIMAP4 | -0.01 (0.08) | -0.01 (0.09) | 0.933 |
| G_HLA_DPA1 | 0.34 (0.11) | 0.25 (0.16) | <0.001 |
| G_LILRB4 | 0.01 (0.10) | -0.06 (0.11) | <0.001 |
| G_SIGLEC9 | -0.05 (0.10) | -0.12 (0.11) | <0.001 |
| G_SLAMF6 | -0.03 (0.10) | -0.09 (0.11) | <0.001 |
| Gamma_delta_T_cell | 0.17 (0.02) | 0.15 (0.04) | <0.001 |
| GRANS_PCA_16704732 | 0.23 (0.02) | 0.21 (0.02) | <0.001 |
| HER2_Immune_PCA_18006808 | 0.16 (0.03) | 0.14 (0.03) | <0.001 |
| HLA | 0.22 (0.07) | 0.17 (0.09) | <0.001 |
| ICR_ACT_SCORE | 0.02 (0.09) | -0.06 (0.12) | <0.001 |
| ICR_INHIB_SCORE | -0.06 (0.10) | -0.13 (0.11) | <0.001 |
| ICR_SCORE | 0.01 (0.09) | -0.07 (0.11) | <0.001 |
| ICS5_score | 0.04 (0.08) | -0.03 (0.11) | <0.001 |
| iDCs | -0.28 (0.14) | -0.33 (0.14) | <0.001 |
| IFIT3 | 0.31 (0.11) | 0.23 (0.14) | <0.001 |
| IFN_21978456 | 0.32 (0.11) | 0.23 (0.15) | <0.001 |
| IFNG_score_21050467 | 0.33 (0.02) | 0.31 (0.03) | <0.001 |
| IgG_19272155 | 0.12 (0.18) | 0.07 (0.18) | 0.001 |
| IGG_Cluster | 0.05 (0.10) | 0.00 (0.11) | <0.001 |
| IGG_Cluster_21214954 | 0.05 (0.10) | 0.00 (0.11) | <0.001 |
| IL12_score_21050467 | 0.03 (0.04) | 0.01 (0.04) | <0.001 |
| IL13_score_21050467 | 0.23 (0.04) | 0.23 (0.05) | 0.413 |
| IL2_score_21050467 | 0.29 (0.02) | 0.28 (0.03) | 0.001 |
| IL4_score_21050467 | 0.32 (0.03) | 0.32 (0.04) | 0.093 |
| IL8_21978456 | 0.02 (0.14) | -0.05 (0.16) | <0.001 |
| Immature__B_cell | 0.06 (0.05) | 0.04 (0.05) | <0.001 |
| Immature_dendritic_cell | 0.23 (0.02) | 0.22 (0.02) | <0.001 |
| Immune_cell_Cluster_21214954 | 0.16 (0.08) | 0.10 (0.10) | <0.001 |
| Immune_NSCLC_score | 0.16 (0.02) | 0.16 (0.02) | 0.006 |
| Inflammation_promoting | 0.03 (0.09) | -0.04 (0.11) | <0.001 |
| Interferon_19272155 | 0.35 (0.10) | 0.26 (0.14) | <0.001 |
| Interferon_Cluster_21214954 | 0.33 (0.08) | 0.27 (0.09) | <0.001 |
| Interferon_ImSig | 0.30 (0.08) | 0.26 (0.08) | <0.001 |
| Interferon_Receptor | 0.33 (0.04) | 0.30 (0.06) | <0.001 |
| Interferons | -0.37 (0.11) | -0.40 (0.09) | <0.001 |
| Interleukins | -0.01 (0.04) | -0.04 (0.05) | <0.001 |
| Interleukins_Receptor | 0.04 (0.03) | 0.01 (0.04) | <0.001 |
| IR7_score | 0.14 (0.09) | 0.07 (0.13) | <0.001 |
| LCK | -0.13 (0.07) | -0.17 (0.09) | <0.001 |
| LCK_19272155 | 0.02 (0.09) | -0.03 (0.09) | <0.001 |
| LIexpression_score | 0.00 (0.11) | -0.06 (0.13) | <0.001 |
| LYM | 0.12 (0.11) | 0.05 (0.11) | <0.001 |
| LYMPHS_PCA_16704732 | 0.35 (0.01) | 0.34 (0.02) | <0.001 |
| Mac_CSF1 | 0.23 (0.03) | 0.20 (0.04) | <0.001 |
| Macrophage | 0.03 (0.04) | 0.02 (0.04) | 0.004 |
| Macrophages | 0.17 (0.07) | 0.10 (0.10) | <0.001 |
| Macrophages_ImSig | 0.16 (0.08) | 0.10 (0.09) | <0.001 |
| Macrophages_M0 | 0.06 (0.07) | 0.01 (0.08) | <0.001 |
| Macrophages_M1 | 0.02 (0.07) | -0.03 (0.08) | <0.001 |
| Macrophages_M2 | 0.05 (0.08) | 0.00 (0.08) | <0.001 |
| MacTh1_cluster | 0.11 (0.09) | 0.04 (0.11) | <0.001 |
| Mast_cell | 0.04 (0.04) | 0.03 (0.04) | <0.001 |
| Mast_cells | -0.23 (0.12) | -0.25 (0.14) | 0.097 |
| Mast_cells_activated | -0.05 (0.06) | -0.09 (0.07) | <0.001 |
| Mast_cells_resting | -0.05 (0.06) | -0.08 (0.06) | <0.001 |
| MCD3_CD8_21214954 | 0.08 (0.05) | 0.14 (0.10) | <0.001 |
| MDACC_FNA_1_20805453 | 0.31 (0.08) | 0.25 (0.10) | <0.001 |
| MDSC | 0.16 (0.08) | 0.11 (0.09) | <0.001 |
| Memory_B_cell | 0.15 (0.03) | 0.15 (0.03) | 0.942 |
| MHC_class_I | 0.46 (0.03) | 0.44 (0.05) | <0.001 |
| MHC_I_19272155 | 0.34 (0.04) | 0.33 (0.04) | 0.008 |
| MHC_II_19272155 | 0.35 (0.09) | 0.26 (0.13) | <0.001 |
| MHC1_21978456 | 0.30 (0.03) | 0.29 (0.03) | 0.097 |
| MHC2_21978456 | 0.35 (0.10) | 0.26 (0.14) | <0.001 |
| Minterferon_Cluster_21214954 | 0.28 (0.07) | 0.22 (0.08) | <0.001 |
| Module11_Prolif_score | 0.22 (0.05) | 0.08 (0.13) | <0.001 |
| Module3_IFN_score | 0.30 (0.10) | 0.22 (0.12) | <0.001 |
| Module4_TcellBcell_score | -0.05 (0.08) | -0.10 (0.08) | <0.001 |
| Module5_TcellBcell_score | 0.03 (0.08) | -0.04 (0.10) | <0.001 |
| Monocyte | 0.23 (0.02) | 0.22 (0.03) | <0.001 |
| Monocytes | 0.01 (0.07) | -0.03 (0.08) | <0.001 |
| Monocytes_ImSig | 0.22 (0.05) | 0.18 (0.06) | <0.001 |
| Monocytic_lineage_MCPcounter | 0.09 (0.09) | 0.02 (0.10) | <0.001 |
| Myeloid_dendritic_cells_MCPcounter | -0.14 (0.07) | -0.15 (0.07) | 0.521 |
| Natural_killer_cell | 0.20 (0.02) | 0.20 (0.02) | 0.003 |
| Natural_killer_T_cell | 0.10 (0.02) | 0.09 (0.03) | <0.001 |
| NaturalKiller_Cell_Cytotoxicity | 0.16 (0.03) | 0.14 (0.03) | <0.001 |
| Neutrophil | -0.14 (0.03) | -0.15 (0.04) | 0.001 |
| Neutrophils | -0.02 (0.06) | -0.05 (0.06) | <0.001 |
| Neutrophils_ImSig | 0.17 (0.03) | 0.16 (0.03) | 0.062 |
| Neutrophils_MCPcounter | -0.05 (0.03) | -0.05 (0.04) | 0.834 |
| NHI_5gene_score | 0.31 (0.07) | 0.24 (0.10) | <0.001 |
| NK_cells | -0.22 (0.07) | -0.28 (0.10) | <0.001 |
| NK_cells_activated | -0.05 (0.07) | -0.09 (0.07) | <0.001 |
| NK_cells_ImSig | -0.23 (0.08) | -0.25 (0.07) | 0.007 |
| NK_cells_MCPcounter | -0.32 (0.06) | -0.32 (0.06) | 0.233 |
| NK_cells_resting | -0.06 (0.07) | -0.09 (0.07) | <0.001 |
| Parainflammation | 0.23 (0.05) | 0.18 (0.07) | <0.001 |
| PD1_data | -0.16 (0.10) | -0.21 (0.11) | <0.001 |
| PD1_PDL1_score | -0.12 (0.08) | -0.15 (0.07) | <0.001 |
| pDCs | -0.05 (0.06) | -0.09 (0.08) | <0.001 |
| PDL1_data | -0.08 (0.08) | -0.09 (0.07) | 0.166 |
| Plasma_cells | 0.02 (0.05) | 0.00 (0.05) | <0.001 |
| Plasma_cells_ImSig | 0.12 (0.13) | 0.10 (0.13) | 0.020 |
| Plasmacytoid_dendritic_cell | 0.27 (0.02) | 0.27 (0.03) | 0.317 |
| Proliferation_ImSig | 0.22 (0.05) | 0.06 (0.15) | <0.001 |
| Regulatory_T_cell | 0.12 (0.06) | 0.07 (0.06) | <0.001 |
| Rotterdam_ERneg_PCA_15721472 | 0.15 (0.02) | 0.12 (0.04) | <0.001 |
| STAT1_19272155 | 0.27 (0.10) | 0.18 (0.14) | <0.001 |
| STAT1_score | 0.16 (0.06) | 0.10 (0.09) | <0.001 |
| T_Cell | 0.17 (0.02) | 0.16 (0.02) | <0.001 |
| T_Cell_cluster | -0.11 (0.08) | -0.15 (0.09) | <0.001 |
| T_cell_co.inhibition | -0.09 (0.08) | -0.11 (0.06) | 0.002 |
| T_cell_co.stimulation | -0.11 (0.06) | -0.15 (0.07) | <0.001 |
| T_cell_infiltration_1 | 0.13 (0.10) | 0.04 (0.14) | <0.001 |
| T_cell_infiltration_2 | 0.02 (0.12) | -0.07 (0.15) | <0.001 |
| T_cell_PCA_16704732 | 0.16 (0.02) | 0.15 (0.02) | <0.001 |
| T_cells_CD4_memory_activated | -0.02 (0.07) | -0.07 (0.08) | <0.001 |
| T_cells_CD4_memory_resting | 0.02 (0.06) | -0.01 (0.06) | <0.001 |
| T_cells_CD4_naive | -0.01 (0.05) | -0.04 (0.06) | <0.001 |
| T_cells_CD8 | -0.01 (0.06) | -0.04 (0.07) | <0.001 |
| T_cells_follicular_helper | 0.04 (0.05) | 0.02 (0.05) | <0.001 |
| T_cells_gamma_delta | -0.04 (0.08) | -0.09 (0.08) | <0.001 |
| T_cells_ImSig | -0.03 (0.08) | -0.07 (0.08) | <0.001 |
| T_cells_MCPcounter | -0.10 (0.06) | -0.14 (0.06) | <0.001 |
| T_cells_regulatory__Tregs | 0.01 (0.06) | -0.02 (0.06) | <0.001 |
| T_follicular_helper_cell | 0.13 (0.03) | 0.12 (0.03) | 0.029 |
| T_helper_cells | 0.32 (0.11) | 0.22 (0.15) | <0.001 |
| TAMsurr_score | 0.11 (0.14) | -0.07 (0.24) | <0.001 |
| TAMsurr_TcClassII_ratio | 0.14 (0.09) | 0.05 (0.13) | <0.001 |
| TcClassII_score | 0.14 (0.09) | 0.07 (0.12) | <0.001 |
| Tcell_21978456 | 0.05 (0.09) | 0.00 (0.10) | <0.001 |
| Tcell_receptors_score | -0.11 (0.11) | -0.17 (0.12) | <0.001 |
| TCRsignalingPathway | 0.14 (0.02) | 0.12 (0.03) | <0.001 |
| Tfh | 0.01 (0.10) | -0.06 (0.11) | <0.001 |
| TGFb_Family_Member | -0.04 (0.04) | -0.04 (0.04) | 0.066 |
| TGFb_Family_Member_Receptor | 0.18 (0.03) | 0.20 (0.04) | <0.001 |
| TGFB_PCA_17349583 | 0.35 (0.05) | 0.33 (0.05) | 0.001 |
| TGFB_score_21050467 | 0.36 (0.03) | 0.35 (0.03) | <0.001 |
| Th1_cells | -0.19 (0.07) | -0.23 (0.08) | <0.001 |
| Th2_cells | 0.00 (0.05) | -0.02 (0.05) | <0.001 |
| TIL | 0.00 (0.07) | -0.04 (0.08) | <0.001 |
| TNBC_B_Cell | 0.20 (0.19) | 0.12 (0.22) | <0.001 |
| TNBC_T_Cell | 0.05 (0.10) | -0.02 (0.12) | <0.001 |
| TNF_Family_Members | 0.01 (0.05) | -0.02 (0.06) | <0.001 |
| TNF_Family_Members_Receptors | 0.08 (0.03) | 0.05 (0.04) | <0.001 |
| Translation_ImSig | 0.49 (0.01) | 0.49 (0.03) | 0.582 |
| Treg | 0.16 (0.02) | 0.15 (0.02) | <0.001 |
| TREM1_data | -0.07 (0.10) | -0.15 (0.13) | <0.001 |
| Troester_WoundSig_19887484 | 0.10 (0.03) | 0.11 (0.03) | 0.114 |
| Type_1_T_helper_cell | 0.11 (0.03) | 0.09 (0.03) | <0.001 |
| Type_17_T_helper_cell | 0.00 (0.02) | -0.01 (0.03) | <0.001 |
| Type_2_T_helper_cell | 0.13 (0.02) | 0.10 (0.03) | <0.001 |
| Type_I_IFN_Reponse | 0.29 (0.10) | 0.22 (0.11) | <0.001 |
| Type_II_IFN_Reponse | 0.09 (0.05) | 0.09 (0.06) | 0.606 |
