# Supplementary material for: Bioinformatics and experimental analysis revealed the cancer-promoting role of NCAPG2 in epithelial ovarian cancer
Source: Front Oncol. 2026 Mar 13;16:1574236. doi: 10.3389/fonc.2026.1574236 (PMC13021424; doi:10.3389/fonc.2026.1574236)
Supplement: Supplementary file 2 [file Table2.docx]

Table 2 qRT-PCR Reaction condition

| Temperature | Time | Number of cycles |
| --- | --- | --- |
| 95℃ | 10 min | 1 |
| 95℃ | 15 s |  |
| 60℃ | 1 min | 40 |
| 95℃ | 15 s |  |
| 60℃ | 1 min |  |
| 95℃ | 15 s | 1 |
