# Supplementary material for: Bioinformatics and experimental analysis revealed the cancer-promoting role of NCAPG2 in epithelial ovarian cancer
Source: Front Oncol. 2026 Mar 13;16:1574236. doi: 10.3389/fonc.2026.1574236 (PMC13021424; doi:10.3389/fonc.2026.1574236)
Supplement: Supplementary file 3 [file Table3.docx]

Table 1 qRT-PCR Reaction system

| Substance | Volume |
| --- | --- |
| SYBR Green Realtime PCR Master Mix | 10 μL |
| Nuclease-free-Water | 6.4 μL |
| Forward primer (10 μM) | 0.8 μL |
| Reverse primer (10 μM) | 0.8 μL |
| cDNA | 2 μL |
| Total volume | 20 μL |
