# Supplementary material for: Bioinformatics and experimental analysis revealed the cancer-promoting role of NCAPG2 in epithelial ovarian cancer
Source: Front Oncol. 2026 Mar 13;16:1574236. doi: 10.3389/fonc.2026.1574236 (PMC13021424; doi:10.3389/fonc.2026.1574236)
Supplement: Supplementary file 4 [file Table4.docx]

**Table** **S4. Sequences (5’-3’)**

| **For qRT-PCR (Primer)** | | |
| --- | --- | --- |
| NCAPG2 | Forward | UUCUCCGAACGUGUCACGUTT |
|  | Reverse | ACGUGACACGUUCGGAGAATT |
| GAPDH | Forward | GGAGTCCACTGGCGTCTTCA |
|  | Reverse | GTCATGAGTCCTTCCACGATACC |
| **For Transfection (siRNA)** | | |
| SI-NCAPG2 | Sense | CCCAGGGUGAAGACAAUAUTT |
|  | Antisense | AUAUUGUCUUCACCCUGGGTT |
| SI-NC | Sense | UUCUCCGAACGUGUCACGUTT |
|  | Antisense | ACGUGACACGUUCGGAGAATT |
